# Nicotinamide Mononucleotide-Producing Probiotic Strain B12-N Attenuates Aging and Cognitive Decline in a *Caenorhabditis elegans* Model of Alzheimer’s Disease: Efficacy and Underlying Mechanisms

**DOI:** 10.3390/foods15203657

**Published:** 2026-10-15

**Authors:** Xueer Wang, Shixiang Wu, Jian He, Ran Wang, Wen Zhao, Zongfeng Zhang, Zhaozhong Zeng, Yufei Zhang, Qiuyue Jiang, Bowen Zhao, Ming Zhang

**Affiliations:** 1 School of Food and Health, Beijing Technology and Business University, Beijing 100048, China; 2 National Technology Innovation Center for Dairy, Hohhot 010100, China; 3 Key Laboratory of Precision Nutrition and Food Quality, Department of Nutrition and Health, China Agricultural University, Beijing 100190, China; 4 Beijing QuantiHealth Technology Co., Ltd., Beijing 100070, China; 5 Inner Mongolia Yili Industrial Group Co., Ltd., Hohhot 010100, China

**Keywords:** Alzheimer’s disease, nicotinamide mononucleotide, *Caenorhabditis elegans*, probiotics

## Abstract

Nicotinamide mononucleotide (NMN) is an active component found in probiotics that may help alleviate Alzheimer’s disease and the effects of aging. The screened NMN-producing strain, *Ligilactobacillus salivarius* B12-N, contained 6.25 μg of NMN per 10^9^ CFU, as determined by LC–MS analysis of bacterial cell extracts. The results indicate that B12-N showed a slightly greater numerical improvement in chemotactic behavior than purified NMN. However, no significant differences were observed between B12-N and NMN in other AD-related or aging-associated phenotypes examined. Transcriptome analysis revealed that 16 pathways were common to both the NMN group and the B12-N group. Notably, the P450 metabolism pathway, longevity regulation pathway, and autophagy pathway were significantly enriched. These findings indicate partially overlapping phenotypic and transcriptomic responses to B12-N and NMN, suggesting that NMN may contribute to some of the biological effects associated with B12-N.

## 1. Introduction

Alzheimer’s disease (AD) is the most prevalent progressive neurodegenerative disorder [1], responsible for approximately 55% to 75% of all dementia cases. With the aging of the global population, the incidence of AD has increased significantly [2]. Projections indicate that the number of individuals affected by AD will reach 78 million by 2030 and 139 million by 2050 [3], imposing a substantial burden on society and the families of patients. Recent research has identified soluble, small oligomeric forms of amyloid-β (Aβ) as the primary neurotoxic agents. Consequently, research efforts have shifted from inhibiting fibril formation to preventing the formation of toxic oligomeric intermediates in the quest to delay the onset of AD and alleviate its symptoms [4,5,6].

Probiotics, which are live microorganisms that confer health benefits on the host when administered in adequate amounts [7], have shown potential in improving AD by maintaining gut microbiota homeostasis [8], reducing inflammatory responses, and mitigating oxidative stress, thereby alleviating cognitive decline [9]. The metabolites produced by probiotics may represent the primary mechanism through which they exert their beneficial effects on AD. Probiotics confer their beneficial effects through the synthesis of advantageous compounds, such as short-chain fatty acids [10] and indole metabolites. These compounds enhance the integrity of the intestinal barrier, contribute to metabolic regulation, and facilitate communication via the gut–brain axis. Fernando [11] demonstrated that in early-stage transgenic AD mice, treatment with sodium butyrate resulted in a 40% reduction in brain Aβ levels and improved cognitive function compared to the control group. Similarly, Zha et al. [12] found that *Bacteroides ovatus* or its metabolite, lysophosphatidylcholine, significantly decreased Aβ plaque burden and ameliorated cognitive impairment in AD model mice. Consequently, there is growing interest in the role of probiotic metabolites in ameliorating AD.

Additionally, research indicates that nicotinamide mononucleotide (NMN) can mitigate the aggregation of toxic oligomers in AD models, making the connection between NMN, AD, and aging a focal point of current research [13]. NMN is a bioactive nucleotide synthesized through the reaction of a nucleoside containing nicotinamide and ribose with a phosphate group [14]. Research has demonstrated that in models of AD, NMN has the potential to ameliorate symptoms associated with AD and delay the aging process by enhancing Aβ clearance and restoring autophagic function [15]. For instance, Yao et al. reported that NMN reduces the formation of amyloid plaques, β-secretase activity, and neuroinflammation, thereby enhancing cognitive function [16]. Similarly, Tarantini et al. observed that NMN ameliorated cerebrovascular and neurovascular coupling impairments in mice, leading to improved cognitive outcomes [17]. Mills et al. further corroborated these findings by demonstrating that prolonged NMN administration can attenuate age-related physiological decline in mice [18]. As a result, the investigation of NMN in the context of AD and aging is garnering increased scholarly interest.

While previous research has identified probiotics as producers of NMN [19,20], further studies are necessary to ascertain if this can effectively ameliorate AD. Probiotics are widely regarded as a significant dietary supplement with numerous health benefits, a claim supported by extensive scientific research [21]. Various microorganisms, including lactic acid bacteria and *Escherichia coli*, have been shown to convert simple substrates into NMN through metabolic pathways [22]. Among these, lactic acid bacteria have emerged as the most promising candidates for NMN production [23]. For instance, Kong et al. employed CRISPR/nCas9 technology to knock out the nadR gene in *Lactococcus* NZ9000, leading to a 61% increase in NMN accumulation [24]. Additionally, probiotics have the potential to influence neuropathology and cognitive function associated with AD by modulating the gut microbiota and its metabolites. Research has demonstrated that specific probiotic strains can alleviate neuroinflammation and inhibit Aβ aggregation through metabolic pathways. For example, studies conducted by Kobayashi and Mehrabadi et al. have shown that *Bifidobacterium breve* A1 and *Bifidobacterium infantis* reduced Aβ, IL-1β, and TNF-α, while enhancing Aβ-induced SOD levels in the hippocampus [25,26]. We therefore hypothesized that NMN-producing probiotic strains may be associated with beneficial effects on AD-related and aging-associated phenotypes.

In this study, strains were isolated from fresh fecal samples collected from healthy, long-lived elderly people in China. The strains were categorized based on their capacity to produce NMN, resulting in the selection of high-producing NMN strains. The effects and mechanisms of the strain in mitigating AD and delaying aging were subsequently evaluated using a nematode model. Specifically, the transgenic *Caenorhabditis elegans* (*C. elegans*) strain CL2355, which expresses human Aβ (Aβ_1–42_) [27], served as the model organism for assessing the AD-alleviating and lifespan-extending effects of the high-producing NMN strain and NMN alone. Furthermore, reactive oxygen species (ROS) levels, autophagy, and age-related physiological functions were also examined. Transcriptomic analysis was further performed to identify molecular pathways potentially associated with the observed phenotypic responses.

## 2. Materials and Methods

### 2.1. Source of Strains and NMN Synthesis Pathway Analysis

All strains used in this study were isolated from fecal samples collected from healthy, long-lived elderly individuals in China. A total of 633 bacterial strains were isolated and identified. The NMN-producing strain, *Ligilactobacillus salivarius* B12-N, was deposited in the China General Microbiological Culture Collection Center (CGMCC, Beijing, China) under accession number CGMCC No. 37828.

Genomic DNA was extracted from the bacterial isolates and subjected to whole-genome sequencing using the Illumina HiSeq 2000 platform. Raw sequencing reads were quality-checked using FastQC, and adapter sequences and low-quality reads were removed using Cutadapt. Host-derived reads were filtered using SOAP2 and MOCAT. The resulting high-quality clean reads were subjected to de novo genome assembly using MEGAHIT. Taxonomic identification was performed using GTDB-Tk (v2.1.1) based on conserved marker genes and genome-scale phylogenetic placement [28].

For analysis of NMN biosynthesis-related pathways, gene prediction and preliminary functional annotation were performed using Prokka (v1.14.6) [29]. KEGG Orthology (KO) annotation was subsequently performed using KofamScan (v1.3.0) [30], and only high-confidence matches meeting the software-defined score thresholds were retained. Candidate genes potentially involved in NMN biosynthesis were identified by mapping the annotated KO terms to the corresponding KEGG metabolic pathways.

### 2.2. Determination of NMN Production by the Strain

#### 2.2.1. Preliminary Screening of NMN-Producing Strains by Fluorimetric Assay

To screen 633 bacterial strains for NMN production capability, a fluorimetric derivatization assay adapted from the method described by Tangrodchanapong [31] was employed in a 96-well plate format. After fermentation, bacterial cultures were centrifuged at 2200× *g* for 10 min, at 4 °C. The cell pellets were washed once with sterile saline and resuspended in an equal volume of deionized water, followed by cell lysis through sonication on ice. After centrifugation at 2200× *g* for 10 min, both the supernatant and the cell lysate supernatant were collected for NMN quantification.

For each reaction in a 96-well plate (250 µL final volume), 69 µL of sample was mixed with 27.7 µL of 20% (*v*/*v*) acetophenone in dimethyl sulfoxide (DMSO) and 27.7 µL of 2 M KOH. The mixture was incubated on ice for 2 min, followed by the addition of 125 µL of 88% (*v*/*v*) formic acid. The plate was then incubated at 37 °C for 10 min. Fluorescence intensity was measured using a microplate reader (e.g., SpectraMax M5, Molecular Devices, San Jose, CA, USA) at an excitation wavelength of 382 nm and an emission wavelength of 445 nm. Strains yielding an NMN concentration above 6.26 × 10^−4^ mM were classified as NMN-producing candidates and selected for further confirmation.

#### 2.2.2. Confirmation and Quantification of NMN by LC-MS

Strains identified as NMN-positive in the preliminary screening were further validated by liquid chromatography–mass spectrometry (LC-MS) [32]. Following fermentation for 12 h, bacterial cells were harvested by centrifugation at 2200× *g* for 10 min at 4 °C. Bacterial cell extracts were prepared as described above and subjected to LC–MS analysis. NMN working standards covering 0.0977–12.5 ng/mL were prepared by serial dilution of an authentic NMN reference standard (CATO Research Chemicals Inc., Guangzhou, China) in deionized water. An external calibration curve was constructed by linear regression of peak area versus concentration. The calibration equation was y = 1.4132 × 10^6^x − 3.4365 × 10^5^, with R^2^ = 0.9947. LC–MS analysis was performed using an Agilent 6545 quadrupole time-of-flight (Q-TOF) LC/MS system (Agilent Technologies, Santa Clara, CA, USA). Chromatographic separation was achieved on an InfinityLab Poroshell 120 Aq-C18 column (4.6 mm × 150 mm, 2.7 μm; Agilent Technologies) maintained at 40 °C.

### 2.3. Preparation of Bacterial Strains and Nematodes

The bacterial strains were cultured under anaerobic conditions in MRS broth at 37 °C for a duration of 12 h. Subsequently, the bacterial cells were harvested through centrifugation and subjected to three washes with phosphate-buffered saline (PBS). The cells were then resuspended in PBS to achieve a concentration of 1 × 10^9^ colony-forming units (CFU) per milliliter [33].

The *C. elegans* strains CL2122, CL2355, BC1292, and DA2123 were obtained from Tianjin University of Science and Technology. *Escherichia coli* OP50, provided by Professor Yanling Hao’s laboratory, served as the standard feed for nematode culture. Following cultivation in LB broth at 37 °C for 12 h, live *E. coli* OP50 cells were collected via centrifugation and washed three times with PBS. These cells were then resuspended in PBS to a concentration of 1 × 10^8^ CFU/mL and subsequently spread onto the surface of nematode growth medium (NGM). Intervening NMN-producing probiotic strain *L. salivarius* B12-N and NMN-content-matched doses were mixed with *E. coli* OP50 for ingestion by nematodes. The nematodes were maintained and propagated on a modified NGM supplemented with 50 mg/L 5-fluoro-2′-deoxyuridine (FUdR) to inhibit oogenesis. Prior to experimentation, all nematodes were synchronized. To prevent contamination and preserve probiotic activity, both OP50 and the intervention strain were added to the culture dishes in concentrated form, with the dishes being replaced every 2–3 days.

### 2.4. Chemotaxis Assay

The chemotaxis assay was executed following the established reference method [31], with slight modifications. The assay utilized 90 mm agar plates composed of 2% agar, 5 mM KHPO_4_, 1 mM CaCl_2_, and 1 mM MgSO_4_. The agar plate was sectioned into four equal quadrants, ensuring that the center of each quadrant was equidistant from the origin. Levamisole hydrochloride (C_11_H_13_ClN_2_S) was applied to designated points to immobilize nematodes migrating towards the quadrant points. Subsequently, the attractant, consisting of 0.1% benzaldehyde diluted in 100% ethanol, and the control solvent (100% ethanol) were administered to the quadrant points. A total of 150 nematodes were placed at the origin and permitted to migrate for a duration of one hour. Five plates per group were utilized as replicates. After one hour, the number of nematodes present in each quadrant was recorded. The chemotaxis index (CI) was calculated using the specified formula:

$$\mathrm{CI}=\frac{\left(N_{\mathrm{attractant}}-N_{\mathrm{control}}\right)}{\left(N_{\mathrm{attractant}}+N_{\mathrm{control}}\right)}$$

N_attractant_ and N_control_ represent the total number of nematodes in the attractant and control zones, respectively.

### 2.5. Thioflavin T Staining of Aβ-Associated Amyloid Aggregates

The ThT assay was conducted in accordance with the reference method [32], incorporating minor modifications. Synchronized L4-stage *Caenorhabditis elegans* strain CL2355 worms were inoculated onto NGM plates containing defined concentrations of NMN and probiotics. Following a 36 h incubation period at 16 °C, the plates were transferred to 25 °C for an additional 36 h. The worms were harvested and washed in M9 buffer. The collected worms were fixed in 4% paraformaldehyde (pH 7.4) at 4 °C for 24 h, then permeabilized in a solution (5% β-mercaptoethanol, 1% Triton X-100 and 125 mM Tris-HCl, pH 7.4) for 24 h at 37 °C, followed by a 2 min immersion in 0.125% ThT and three washes. ThT fluorescence was imaged using the FITC channel of a Leica DM6B upright microscope (Leica Microsystems CMS GmbH, Wetzlar, Germany). The fluorescence signal was used to assess the burden of ThT-positive amyloid-like aggregates in the CL2355 worms.

### 2.6. ROS Assay

The ROS assay was performed following a reference method [34], with slight modifications. Intracellular ROS levels were assessed using 2′,7′-dichlorodihydrofluorescein diacetate (DCFH-DA) at a final concentration of 1 μM. Synchronized L4-stage CL2355 nematodes were placed on NGM plates supplemented with specific concentrations of NMN and probiotics. Following an initial incubation period of 36 h at 16 °C, the cultures were subsequently transferred to 25 °C for an additional 36 h. The nematodes were then washed with M9 buffer and incubated in the dark with 1 μM DCFH-DA for 2 h. After staining, the worms were washed with M9 buffer and mounted on 2% agarose pads. Fluorescence images were acquired using a Leica DM6 B upright fluorescence microscope equipped with a 10× objective, with an exposure time of 512.22 ms and a gain setting of 4.0. Identical imaging settings were maintained for all experimental groups. Fluorescence intensity was quantified using ImageJ software (version 1.54g; National Institutes of Health, Bethesda, MD, USA). The mean fluorescence intensity of the model group was set to 1.0, and the fluorescence intensity of each sample was expressed relative to this value. n = 15 worms per group, and the experiment was independently repeated three times.

### 2.7. Autophagy-Related Assay

The autophagy assay was conducted in accordance with a reference method [35], with minor adaptations. In this investigation, the transgenic *C. elegans* reporter strains DA2123 (LGG-1::GFP) and BC12921 (SQST-1::GFP) were used to assess autophagy-related changes. Synchronized L4-stage DA2123 or BC12921 worms were placed on NGM plates containing specified concentrations of NMN and probiotics. After a 36 h incubation at 16 °C, the plates were transferred to 25 °C for a further 36 h. The worms were harvested using M9 buffer and transferred to 2% agarose pads. Fluorescence images of the worms were acquired using the FITC channel of a Leica DM6B upright microscope. Analysis was performed using ImageJ software. For the DA2123 worms, the number of LGG-1::GFP -positive puncta in the seam cells was quantified. The fluorescence intensity of SQST-1::GFP in the BC12921 nematodes was measured. A total of 15 worms per group were analyzed in each independent experiment, and the experiment was independently repeated three times.

### 2.8. Lifespan Measurement

For the lifespan assay, a previously established protocol was followed with minor modifications [36]. The temperature regimen was selected according to the temperature-sensitive characteristics of the CL2355 strain. CL2355 carries a temperature-sensitive smg-1 background and is commonly maintained at a lower temperature to suppress premature expression of the neuronal human Aβ_1–42_ transgene [37,38]. Synchronized CL2355 worms were initially maintained on NGM plates containing the indicated concentrations of NMN or probiotic treatments at 16 °C for 36 h. The plates were then transferred to 25 °C for 36 h to induce neuronal Aβ_1–42_ expression, consistent with previously reported protocols for the CL2355 model [38]. Following the induction period, the worms were maintained at 20 °C for the remainder of the lifespan assay, as 20 °C is a standard condition for *C. elegans* lifespan analysis and avoids the additional influence of prolonged high-temperature exposure on longevity [36]. For each independent lifespan experiment, 120 worms per treatment group were distributed across 12 NGM plates, with 10 worms per plate. The complete lifespan assay was independently repeated three times. Survival was monitored daily until all worms had died. Worms were considered dead when they failed to respond to gentle mechanical stimulation with a platinum wire.

### 2.9. Pharyngeal Pumping Rate

The pharyngeal pumping rate was quantified according to the methodology described by Zhao et al. [39], with assessments conducted on days 12 and 20. Nematodes were collected and rinsed using M9 buffer. The pharyngeal pumping rate was determined by observing the number of contractions per 60 s under a microscope at specified time intervals.

### 2.10. Assessment of Motility

The motility of *C. elegans* was also evaluated on days 12, 16, 20, and 24, with classifications based on a previously established method [40]. Class A worms exhibited normal spontaneous movement; Class B worms showed impaired spontaneous locomotion but were able to move upon mechanical stimulation; Class C worms displayed only slight head or tail movement following stimulation; and Class D worms showed no detectable movement. This classification was used as a semi-quantitative measure of age-related decline in locomotor function.

### 2.11. Oil Red O Staining

Oil Red O staining (Aladdin, Shanghai, China) was conducted in accordance with previously established protocols [41]. Following the completion of the intervention, nematodes were collected through washing with M9 buffer. A 1% Oil Red O solution was prepared by dissolving Oil Red O in isopropanol (Aladdin, Shanghai, China) containing 2% Triton X-100. The nematodes were fixed for 20 min, stained, rinsed with M9 buffer, and subsequently placed on a 2% agarose gel pad. After staining, worms were anesthetized with 10 mM levamisole and mounted on 2% agarose pads. Representative images were acquired under brightfield illumination using a Leica DM6 B upright microscope. Oil Red O staining was quantified using ImageJ software, and the relative staining intensity was used as an indicator of neutral lipid accumulation. Background staining was subtracted during image analysis [42].

### 2.12. Transcriptomic Analysis

On day 5 of the intervention, according to predefined groups, CL2355 strain were harvested via washing with M9 buffer, followed by total RNA extraction utilizing QIAzol Lysis Reagent (Qiagen, PL Venlo, The Netherlands, Cat. No. 79306). Each experimental group consisted of three biological replicates, with each replicate containing more than 1000 nematodes per replicate. Library preparation and RNA sequencing were performed by Majorbio (Shanghai, China). Libraries were prepared using the Illumina^®^ Stranded mRNA Prep Kit (Illumina, San Diego, CA, USA) and sequenced on a NovaSeq Xplus (Illumina, San Diego, CA, USA). Quality control of the sequencing data was performed using fastp (Shifu Chen. fastp 1.0). Subsequently, differential gene expression analysis and Gene Ontology (GO) and Kyoto Encyclopedia of Genes and Genomes (KEGG) pathway enrichment analyses were conducted using the Majorbio Cloud Platform (https://www.majorbio.com/). DEGs were identified using thresholds of FDR < 0.01 and |log2(fold change)| > 1.5.

### 2.13. Statistical Analysis

Data are presented as mean ± standard deviation (SD), unless otherwise indicated. Statistical analyses were performed using GraphPad Prism software (version 10.4.2; GraphPad Software, San Diego, CA, USA). Survival data were analyzed using Kaplan–Meier survival analysis, and differences between survival curves were assessed using the log-rank (Mantel–Cox) test. The percentage increase in mean lifespan was calculated relative to the model group as [(mean lifespan of the treatment group − mean lifespan of the model group)/mean lifespan of the model group] × 100%.

For continuous outcomes measured at a single time point, differences among groups were analyzed using one-way analysis of variance (ANOVA) followed by Tukey’s multiple-comparison test. For pharyngeal pumping rate measured at different ages, treatment groups were compared separately at each assessment time point using one-way ANOVA followed by Tukey’s multiple-comparison test. Motility data were treated as ordinal categorical data and are presented descriptively as the percentages of worms classified into Classes A–D at each assessment time point; no one-way ANOVA was applied to these data. A *p* value < 0.05 was considered statistically significant.

## 3. Results

### 3.1. NMN Production Pathways in Probiotics Based on Genomic Data

**Figure 1:**
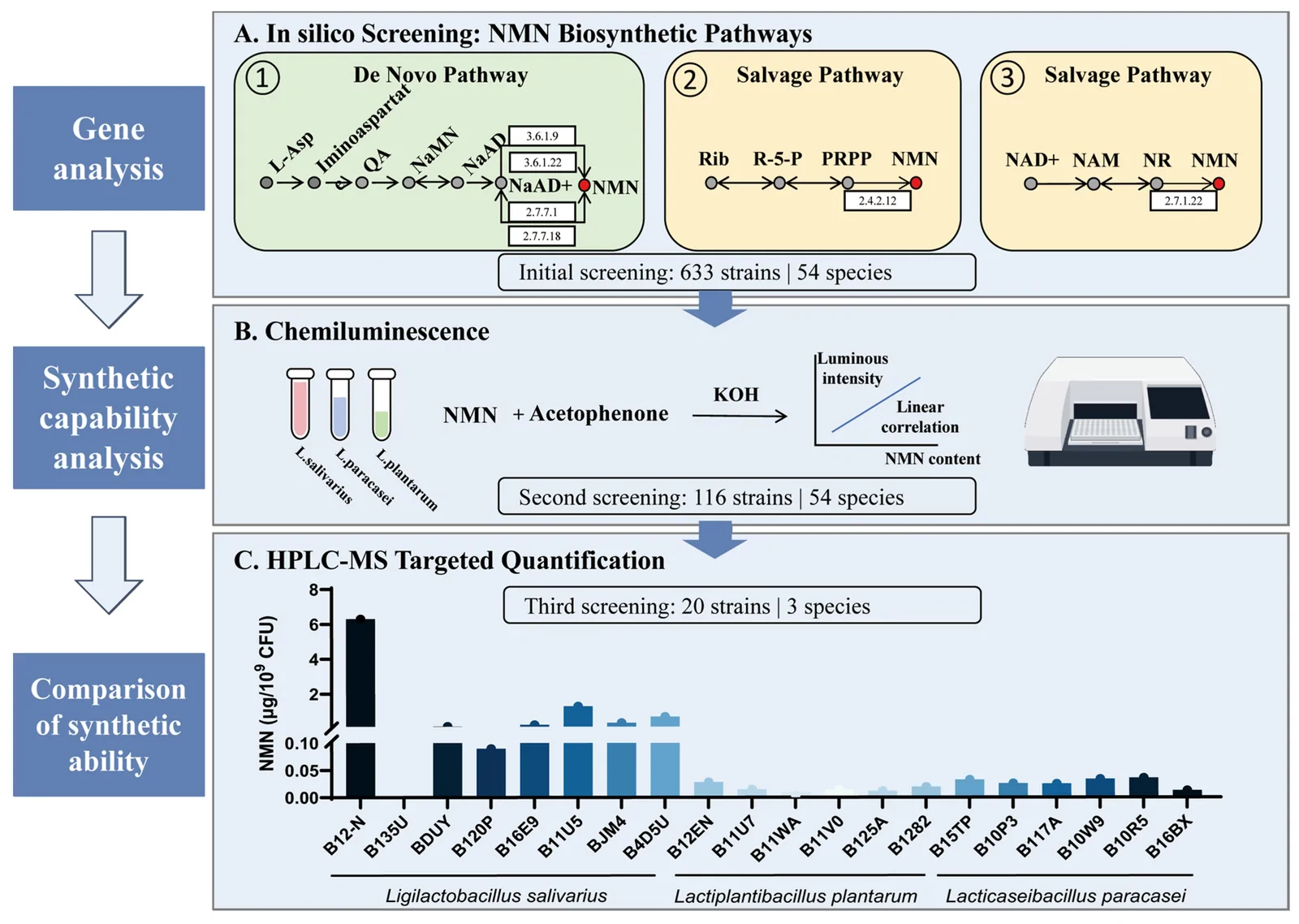
Screening of NMN-producing strains. (**A**) Three core pathways for NMN synthesis. (**B**) Screening of NMN-producing strains by chemiluminescence. (**C**) NMN content produced by strain.

According to the KEGG database map00760 pathway, we identified three main pathways for NMN synthesis (Figure 1), including: pathway 1: L-Aspartate→Iminoaspartate→Quinolinate→Nicotinate D-ribonucleotide↔Deamino-NAD+→NAD+→NMN; pathway 2: Rib↔R-5-P↔PRPP→NMN; and pathway 3: NAD+→Nicotinamide↔N-Ribosyl-nicotinamide→NMN. Pathway 1 belongs to the de novo synthesis pathway, while pathways 2 and 3 belong to the salvage synthesis pathway.

**Figure 2:**
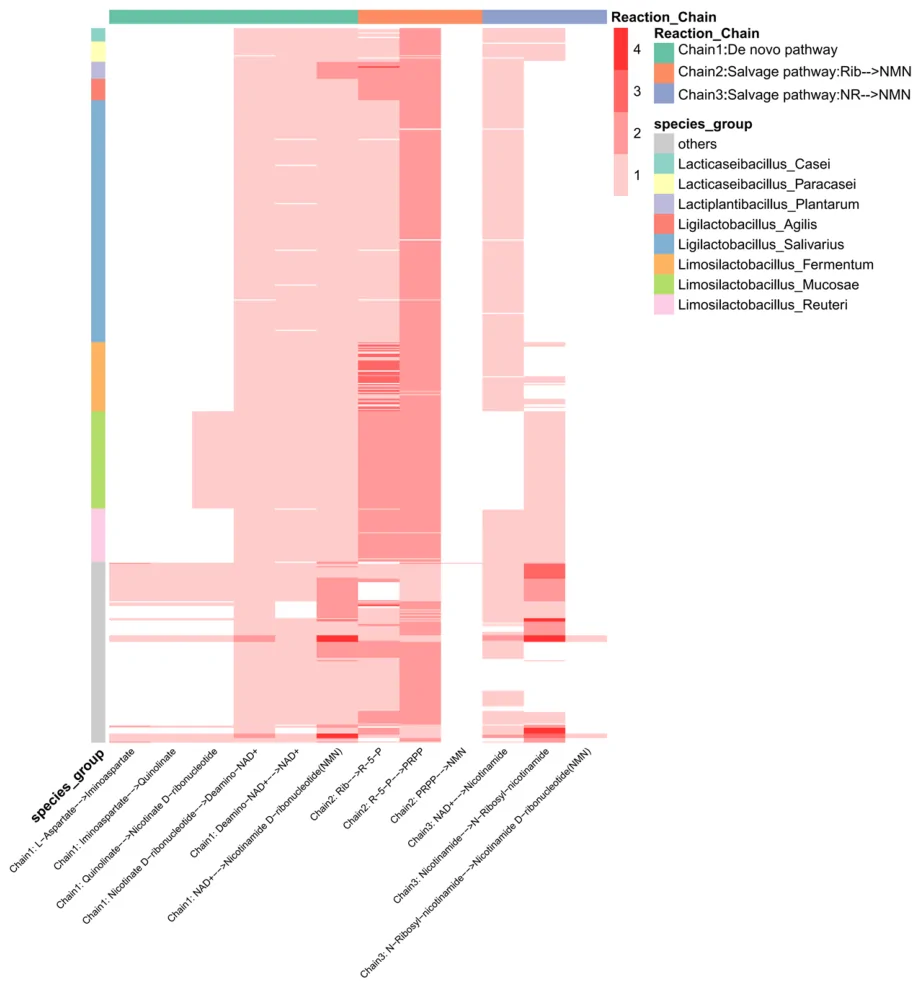
A heatmap of the number of KO copies corresponding to the enzymes catalyzing reactions in the strain. The columns represent the enzymes involved in the catalytic reactions, and the rows represent the strains. The shade of color corresponds to the number of KO copies of the enzyme involved in the catalytic reaction. The row labels indicate the strain, and the column labels indicate the reaction pathway to which they belong.

Based on the core screening strategy that the KO copy number corresponding to the last step enzyme in the three reaction chains is non-zero, we screened 633 strains from our strain library, divided into 54 species, mainly including 214 strains of *Lgb. salivarius*, 87 strains of *Limosilactobacillus mucosae*, 61 strains of *Limosilactobacillus fermentum*, 47 strains of *Limosilactobacillus reuteri*, 19 strains of *Ligilactobacillus agilis*, 15 strains of *Lactiplantibacillus plantarum*, and 18 strains of *Lacticaseibacillus paracasei*. Based on the number of major gene copies of the last step enzyme in NMN synthesis (Figure 2), we found that almost all lactic acid bacteria in this study synthesize NMN mainly through pathway 1, with only a few strains capable of synthesizing NMN through pathways 2 and 3.

### 3.2. Screening of NMN-Producing Probiotic Strains

From an initial pool of 633 candidate strains, the NMN content synthesized by each strain was verified through a fluorimetric NMN assay. Through this screening process, 116 strains capable of NMN synthesis were identified (Figure 1B). Subsequently, 20 probiotic strains were selected for quantitative analysis using liquid chromatography–mass spectrometry (LC–MS), based on criteria such as species classification, yield potential, inherent fermentation performance, and applicability for further development. The selected strains included eight *Lgb. salivarius*, six *Lcb. paracasei* and six *Lpb. plantarum*. As shown in Figure 1C and Supplementary Figure S2, the analysis revealed that *Limosilactobacillus salivarius* exhibited significantly higher intracellular NMN accumulation levels compared to the other strains tested, with the B12-N strain demonstrating the highest NMN content at 6.25 μg/10^9^ CFU. Therefore, the *Lgb. salivarius* B12-N strain was selected as a high-NMN-producing probiotic strain for further experimental research, and a purified NMN dose matched to the measured NMN content of 10^9^ CFU B12-N (6.25 μg) was included as an NMN-content-matched reference treatment. The *Lgb. salivarius* B135U strain, as a low-NMN-producing strain, served as a negative control strain in chemotaxis experiments and autophagy level detection.

### 3.3. NMN and Probiotics Ameliorated Aβ-Associated Behavioral Dysfunction in a C. elegans Model of AD

#### 3.3.1. NMN and Probiotics Ameliorated Chemotaxis Deficits in *C. elegans* CL2355

**Figure 3:**
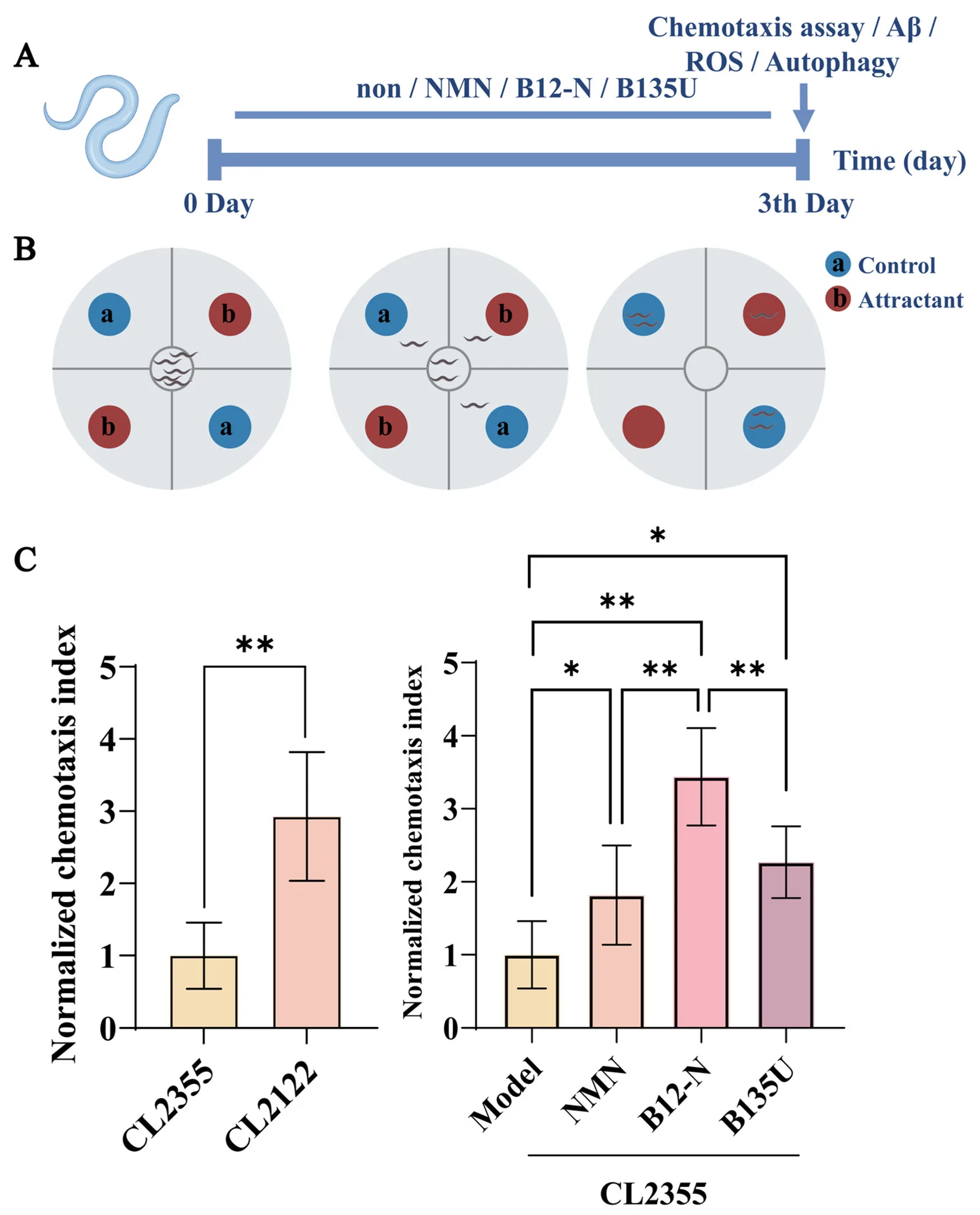
The effects of NMN and probiotics on the chemotaxis of *C. elegans* CL2355. (**A**) An experimental flowchart. (**B**) A schematic diagram of the chemotaxis assay. (**C**) An assessment of chemotactic behavior in the neuron-expressing Aβ nematode strain CL2355. The chemotaxis index (CI) was measured in CL2355 nematodes following treatment with B12-N (1 × 10^9^ CFU), B135U (1 × 10^9^ CFU) or an NMN-content-matched dose of purified NMN (6.25 μg, corresponding to the NMN content measured in 1 × 10^9^ CFU of B12-N). CL2122 worms served as the non-Aβ-expressing control strain. n = 150 worms per group (* *p* < 0.05, ** *p* < 0.01).

The *C. elegans* CL2355 strain is a transgenic nematode model that expresses Aβ in neuronal cells, which is extensively utilized to explore the potential neuroprotective effects of inhibiting Aβ oligomerization against Aβ-induced neurotoxicity. Initially, we examined the influence of B12-N, B135U and content-matched dose of NMN (6.25 μg/10^9^ CFU) on the chemotactic performance of CL2355 nematodes. A chemotaxis impairment model was developed through temperature-controlled transformation, revealing that the CI of CL2355 worms was significantly reduced compared to CL2122 worms, demonstrating a pronounced decline in chemotactic behavior (Figure 3C), thereby confirming the successful establishment of the model. The CI values in the NMN and B12-N treatment groups were significantly elevated relative to the untreated control group, indicating a substantial enhancement in chemotaxis towards the odorant benzaldehyde (Figure 3C). In particular, the worms administered with B12-N demonstrated the most significant enhancement in chemotaxis, surpassing the performance of those treated with NMN alone (*p* < 0.01). And the improvement in chemotaxis of B135U was significantly lower than that with B12-N. Given that the NMN content in the B12-N bacteria was comparable to that in the NMN intervention group, we hypothesize that the neuroprotective effect of B12-N and its ability to alleviate chemotaxis defects are to some extent associated with NMN, whilst the bacteria also contain other neuroprotective compounds.

#### 3.3.2. NMN and B12-N Reduce Aβ-Associated Amyloid Aggregates and ROS Levels in *C. elegans* CL2355

**Figure 4:**
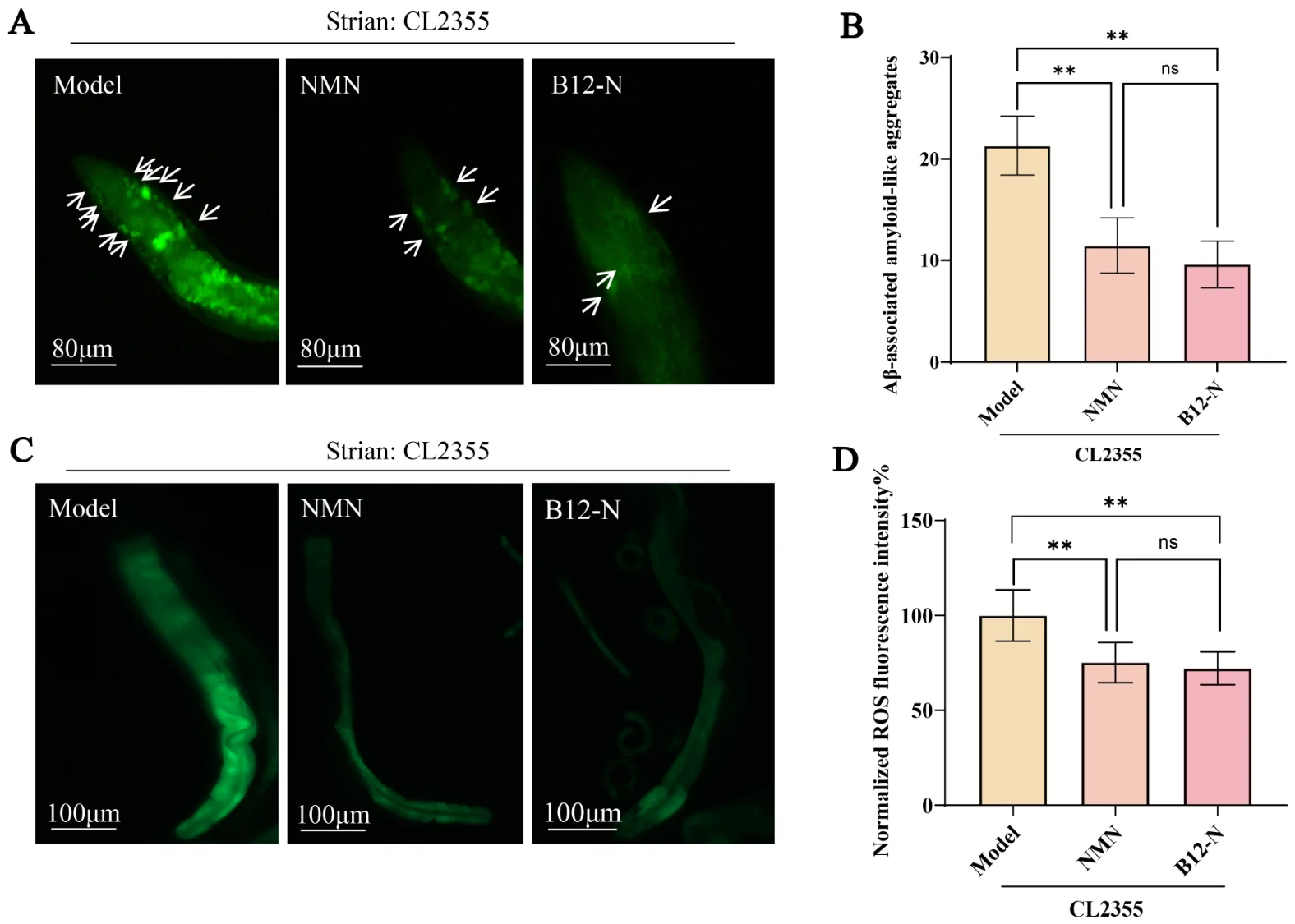
Aβ-associated amyloid aggregates and ROS levels in CL2355 nematodes following NMN and probiotic intervention. (**A**) Representative fluorescence images of Aβ-associated amyloid aggregates in CL2355 nematodes. (**B**) The quantification of Aβ-associated amyloid aggregates in CL2355 nematodes, n = 15 worms per group. The arrows indicate representative prominent ThT-positive amyloid-like aggregates and do not represent all aggregates included in the quantitative analysis. All detectable ThT-positive aggregates within the defined head/anterior region were included in the quantification. (**C**) A representative image of ROS fluorescence intensity in CL2355 nematodes. (**D**) The fluorescence intensity of ROS in CL2355 nematodes, n = 15 worms per group. The fluorescence microscopy channel used was the FITC channel. The data are presented as mean ± SD. The experiments were independently repeated three times (ns, not significant, ** *p* < 0.01).

Subsequently, we quantified the Aβ-associated amyloid aggregates in the heads of CL2355 to evaluate whether the inhibitory effects of NMN and probiotics on Aβ oligomerization could mitigate amyloidosis. The findings revealed the presence of Aβ-associated amyloid aggregates in CL2355. However, the number of Aβ-associated amyloid aggregates in the heads of CL2355 worms within the NMN and B12-N groups were significantly diminished (*p* < 0.01, Figure 4B). Even though worms given B12-N had the lowest Aβ-associated amyloid aggregates, there was no notable difference when compared to the NMN groups (*p* > 0.05).

Oxidative stress is closely associated with Aβ toxicity and AD-related pathology [43,44]. Numerous studies have established a correlation between oxidative stress, Aβ toxicity, and AD. In this study, we measured ROS levels in CL2355 worms to evaluate the effects of NMN and probiotic treatments on oxidative stress. The results demonstrate that CL2355 worms treated with NMN and B12-N exhibited a significant reduction in ROS levels (*p* < 0.01, Figure 4D). Consistent with the pattern observed for Aβ-associated amyloid aggregates, B12-N-treated worms exhibited the lowest numerical ROS levels; however, the difference between the B12-N and NMN groups was not statistically significant (*p* > 0.05).

#### 3.3.3. NMN and Probiotics Modulated Autophagy-Related Activity of *C. elegans* Strains DA2123 and BC12921

**Figure 5:**
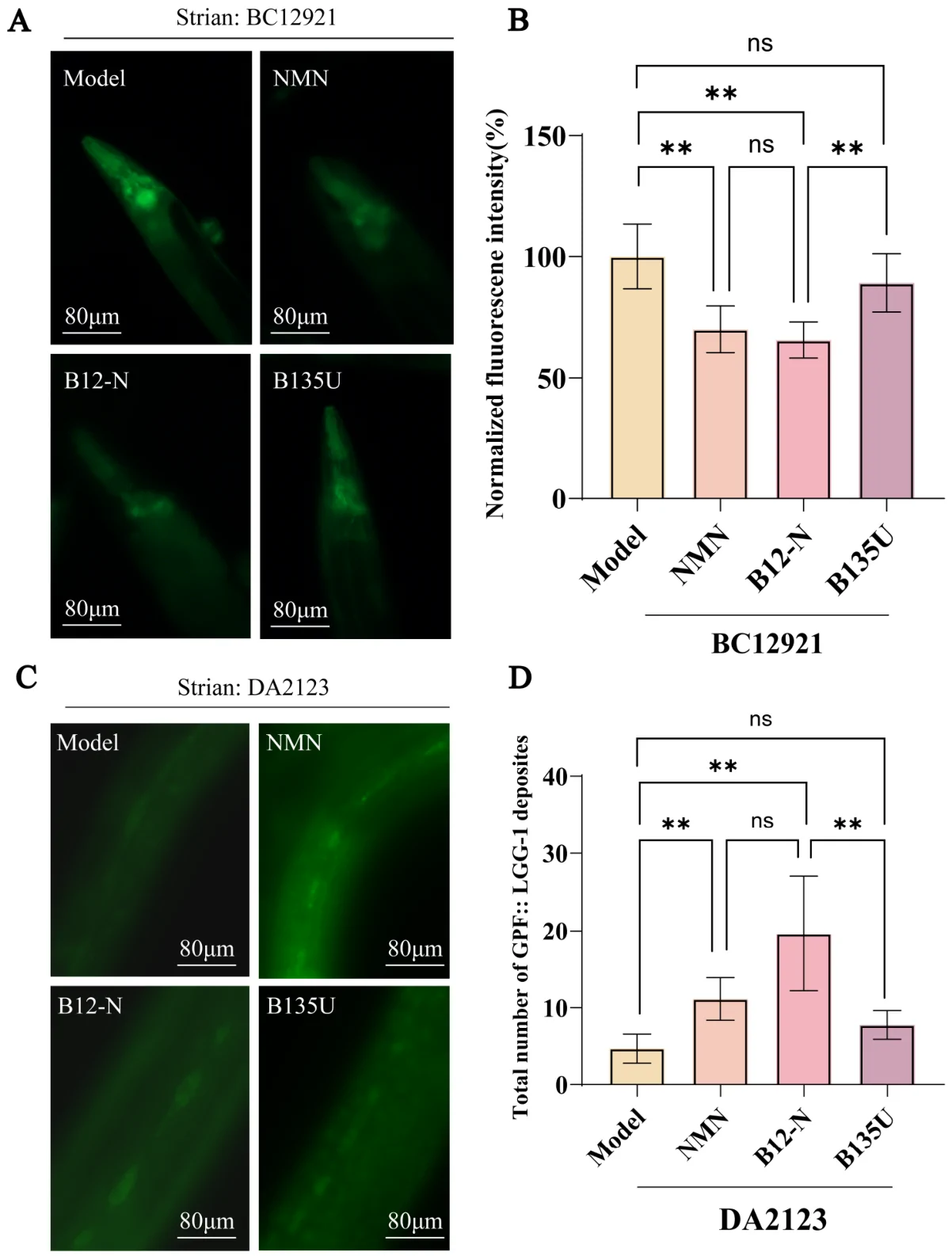
Autophagy-related changes in BC12921 and DA2123 nematodes following NMN and probiotic intervention. (**A**) A representative image of *SQSTM1/p62* protein fluorescence in BC12921 nematodes. (**B**) The GFP intensity of *SQSTM1/p62* protein expression in BC12921 nematodes, n = 15 worms per group. (**C**) A representative image of LGG-1::GFP fluorescence in the seam cells of DA2123 nematodes. (**D**) The mean number of LGG-1::GFP puncta in seam cells of DA2123 nematodes, n = 15 worms per group. The fluorescence microscopy channel used was the FITC channel. The experiments were independently repeated three times; the data are presented as mean ± SD. (ns, not significant, ** *p* < 0.01).

Impaired autophagy is a critical characteristic of AD and further exacerbates its progression. To investigate the effects of NMN and probiotics on autophagic function, we utilized the *C. elegans* BC12921 model, which expresses a SQSTM1/p62 fusion protein as an autophagy marker. Reduced SQST-1/p62 accumulation is generally consistent with increased autophagic degradation [45,46]. NMN and B12-N treatment significantly reduced SQST-1::GFP accumulation in the head region of BC12921 worms (*p* < 0.01, Figure 5B), with no significant difference between the two treatment groups. However, no significant decrease was observed in the B135U group. Because SQST-1/p62 is an autophagic substrate that is degraded through the autophagy–lysosomal pathway, its reduced accumulation is generally consistent with enhanced autophagic degradation. These findings therefore suggest that both NMN and B12-N may modulate autophagy-related activity and promote the clearance of autophagic substrates [47,48].

The DA2123 strain serves as another established transgenic reporter strain for monitoring the autophagy process, characterized by the expression of an LGG-1::GFP fusion protein driven by the lgg-1 promoter [49]. During autophagy, the LGG-1::GFP fusion protein redistributes and localizes to form punctate structures that represent pre-autophagosomes and autophagosomal components. Consequently, LGG-1::GFP serves as a reliable marker for detecting autophagy in *C. elegans* [50,51]. An increased number of LGG-1::GFP puncta may reflect either increased autophagosome formation or impaired autophagosome degradation. The experimental results demonstrate a significant increase (*p* < 0.01) in the number of LGG-1::GFP foci within the seam cells of DA2123 worms treated with NMN and B12-N (Figure 5D). However, no significant increase was observed in the B135U group. Notably, the increase in LGG-1::GFP puncta was comparable between the B12-N and NMN groups, with no statistically significant difference observed between the two treatments.

Taken together, the increase in LGG-1::GFP puncta and the reduction in SQST-1::GFP accumulation observed following both NMN and B12-N treatment are consistent with enhanced autophagic activity in nematodes. The comparable autophagy-related effects observed in the NMN and B12-N groups suggest that the two interventions may modulate autophagy to a similar extent under the present experimental conditions. However, there was no significant difference between the B135U group and the model group. Given that the NMN content measured in B12-N was nominally matched to the purified NMN treatment, these findings are consistent with the possibility that NMN may contribute, at least in part, to the autophagy-related effects of B12-N.

### 3.4. NMN and Probiotics Alleviate Aging Characteristics in a C. elegans CL2355

#### 3.4.1. NMN and Probiotics Extended the Lifespan of *C. elegans* CL2355

**Figure 6:**
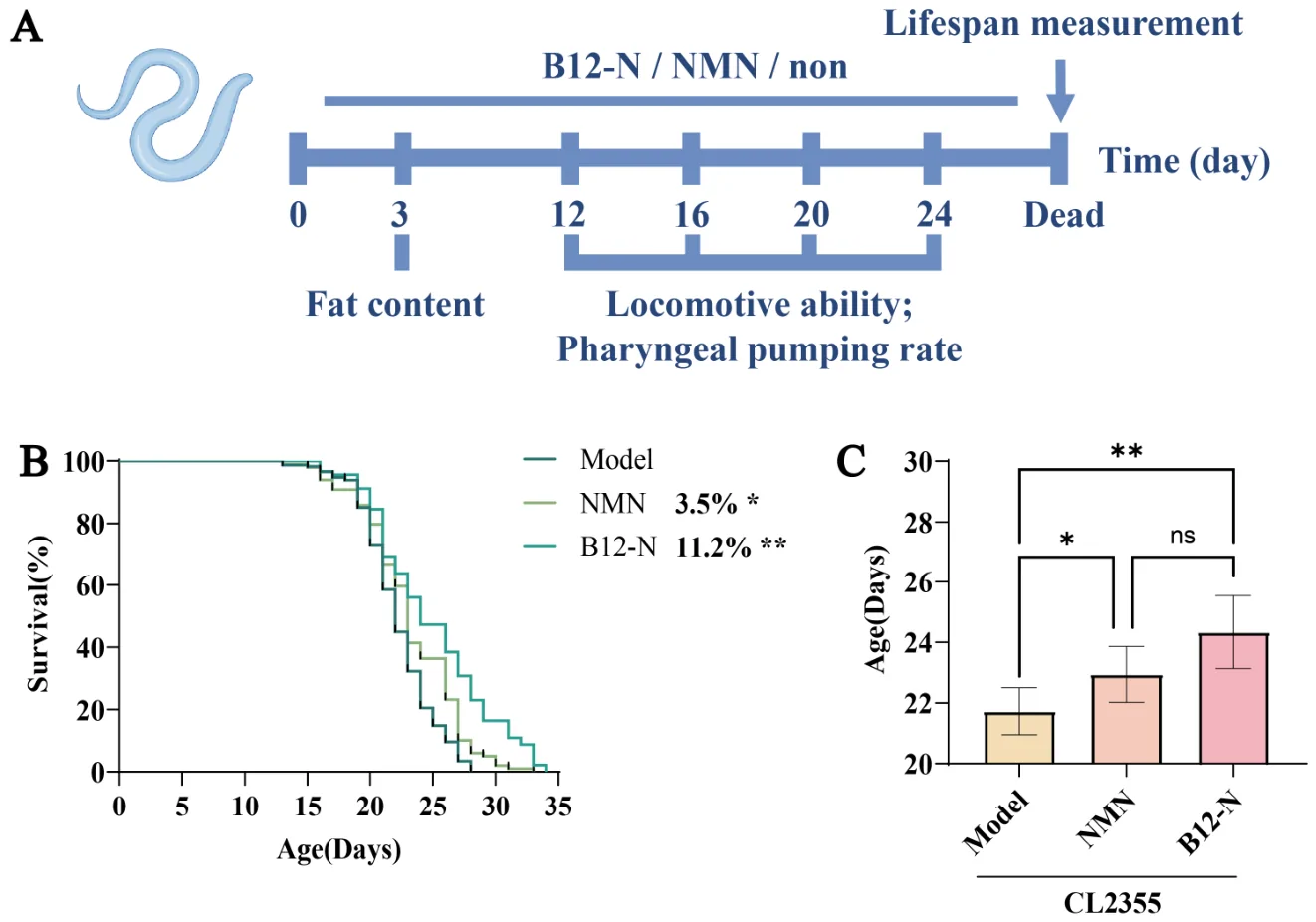
The effects of NMN and probiotics on the lifespan of *Caenorhabditis elegans* CL2355. (**A**) An experimental timeline showing the NMN and B12-N interventions and the assessment times for lifespan, pharyngeal pumping rate, motility, and neutral lipid accumulation. (**B**) The Kaplan–Meier survival curves of CL2355 worms in the different intervention groups. For each independent lifespan experiment, 120 worms per group were distributed across 12 replicate NGM plates (10 worms per plate), and the experiment was independently repeated three times. Survival curves were analyzed using the Kaplan–Meier method and compared using the log-rank (Mantel–Cox) test. (**C**) The mean lifespan of CL2355 worms in the different intervention groups. The mean lifespan was calculated from the individual survival times of worms within each independent experiment, and the reported values were obtained from three independent lifespan experiments. (ns, not significant, * *p* < 0.05, ** *p* < 0.01).

Previous studies have reported that neuronal degeneration in nematodes impairs their development, underscoring the close relationship between aging and neurodegenerative diseases. In this study, we investigated the impact of NMN and probiotic supplementation on the lifespan of nematodes. The findings indicated that NMN and B12-N significantly prolonged the average lifespan of CL2355 nematodes (Figure 6B,C). Specifically, the NMN group exhibited an average lifespan of 22.64 days, reflecting a 3.5% increase compared to the control group, which had an average lifespan of 21.88 days. The B12-N group demonstrated an average lifespan extension to 24.34 days, corresponding to an 11.2% increase.

#### 3.4.2. NMN and B12-N Improve Pharyngeal Pumping and Age-Related Functional Phenotypes in CL2355 *C. elegans*

**Figure 7:**
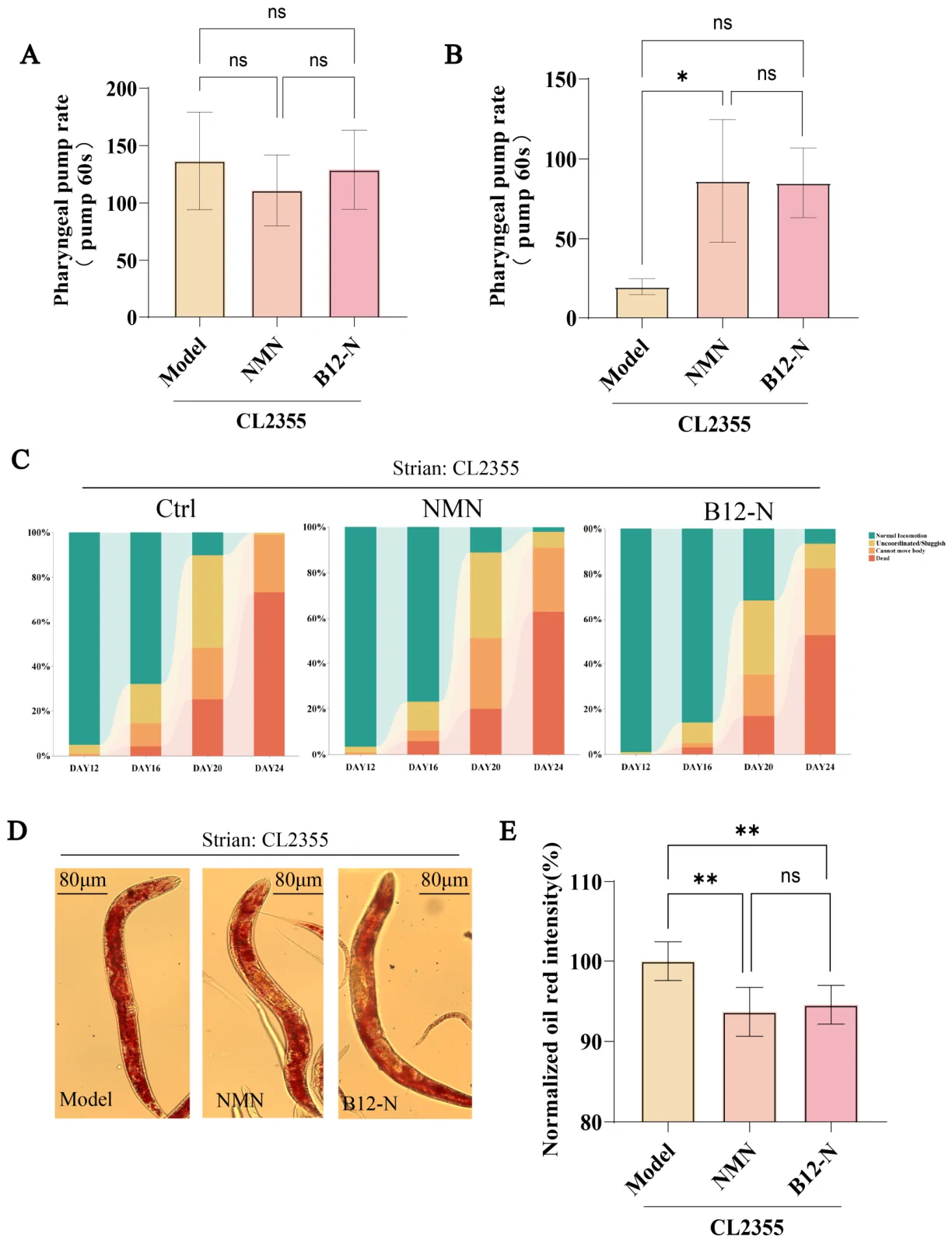
Pharyngeal pump rate, motility and lipid accumulation in CL2355 nematodes following NMN and probiotic intervention. (**A**) Pharyngeal pump frequency (pumps/min) in CL2355 nematodes on day 12, n = 15 worms per group. (**B**) Pharyngeal pump frequency (pumps/min) in CL2355 nematodes on day 20, n = 15 worms per group. (**C**) Proportion of CL2355 nematodes classified into motility categories A, B, C and D on days 12, 16, 20 and 24, n ≥ 100 worms per group. (**D**) Representative images of Oil Red O staining reflecting neutral lipid accumulation in CL2355 nematodes on day 3. (**E**) Relative Oil Red O staining intensity in CL2355 nematodes on day 3, n = 15 worms per group. (ns, not significant, * *p* < 0.05, ** *p* < 0.01).

The pharyngeal pump rate, a crucial indicator of nematode health, was assessed. On day 12, no significant differences in pharyngeal pumping frequency were observed between the groups (Figure 7A), suggesting that the supplements did not significantly affect food intake, feeding preferences, or interfere with the pharyngeal pumping rate. By day 20, the pharyngeal pumping rate of CL2355 nematodes in the model group had declined to an average of 20 beats per minute. In contrast, the NMN and B12-N groups maintained pharyngeal pumping frequencies at 86 and 85 beats per minute, representing increases by 331% and 325% compared with the control group (Figure 7B). These findings indicate that NMN and B12-N attenuated the age-associated decline in pharyngeal pumping observed in CL2355 nematodes.

Previous studies have documented that nematodes display age-associated declines in locomotor function, including reduced motility [52]. Therefore, this study evaluated the impact of NMN and probiotics on the motility of CL2355 nematodes at 12, 16, 20, and 24 days of adulthood. The distribution of motility categories shifted toward lower-activity classes with advancing age (Figure 7C). Notably, on days 16 and 20, nematodes in the NMN and B12-N treatment groups showed a greater proportion of worms remaining in Class A on days 16 and 20. These observations suggest that NMN and probiotics may attenuate the age-related decline in locomotor function in CL2355 nematodes.

Additionally, lipid homeostasis is closely associated with aging and longevity in *C. elegans*. Excessive neutral lipid accumulation and dysregulated lipid metabolism may contribute to lipotoxicity, mitochondrial dysfunction, and oxidative stress. Therefore, Oil Red O staining was performed to evaluate neutral lipid accumulation in *C. elegans* as an aging-associated metabolic phenotype. B12-N treatment significantly reduced lipid accumulation (*p* < 0.01, Figure 7E), suggesting that the lifespan-extending effect of B12-N was accompanied by an improvement in age-associated lipid homeostasis. These findings suggest that B12-N and an equivalent amount of NMN can delay the phenotypes associated with aging in CL2355 nematodes, thereby increasing their average lifespan.

### 3.5. Effects of NMN and B12-N Treatment on the Transcriptional Profiles of CL2355 Strain

**Figure 8:**
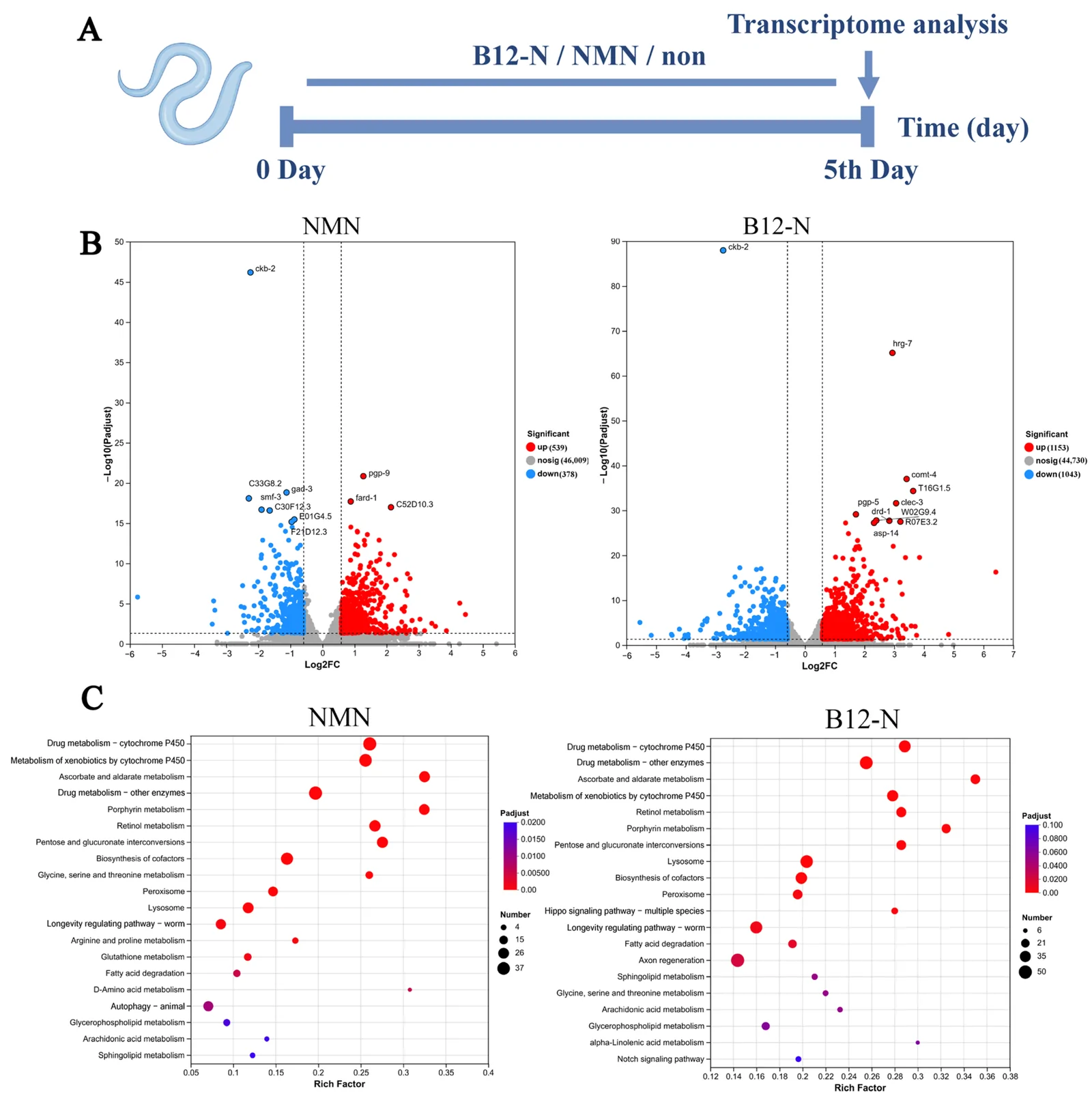
The transcriptomic results from CL2355 nematodes following NMN and probiotic intervention. (**A**) The timing of NMN and probiotic interventions as well as the timing of transcriptomic sampling. (**B**) A volcano plot of differentially expressed genes in the NMN and B12-N groups compared to the control group. (**C**) A KEGG enriched pathway bubble plot for CL2355 nematodes in NMN and the B12-N group compared to the control group.

As illustrated in Figure 8A, on the fifth day of the intervention, the CL2355 strain from the control group, the NMN group, and the B12-N group underwent three rinses with M9 buffer to ensure the acquisition of clean worms, which were subsequently processed for transcriptomic analysis as previously described. The NMN group and the B12-N group exhibited 917 and 2196 differentially expressed genes, respectively. Within the NMN group, 539 genes were up-regulated and 378 were down-regulated, whereas in the B12-N group, 1153 genes were up-regulated and 1043 were down-regulated, as depicted in Figure 8B. Thus, B12-N treatment was associated with a broader transcriptomic response than NMN treatment under the conditions examined, as reflected by the larger number of DEGs.

KEGG pathway enrichment analysis was conducted on the differentially expressed genes, and Figure 8C presents the top 20 enriched pathways in nematodes from both the NMN and B12-N groups. Sixteen enriched pathways were shared between the two intervention groups, indicating partial overlap in their transcriptomic responses. This overlap is consistent with the possibility that NMN may contribute to some of the molecular responses associated with B12-N treatment. Notably, the P450 metabolism pathway, longevity regulation pathway, and autophagy pathway were significantly enriched, and are clearly associated with AD and longevity, with 37, 23 and 22 genes enriched in each, respectively.

**Figure 9:**
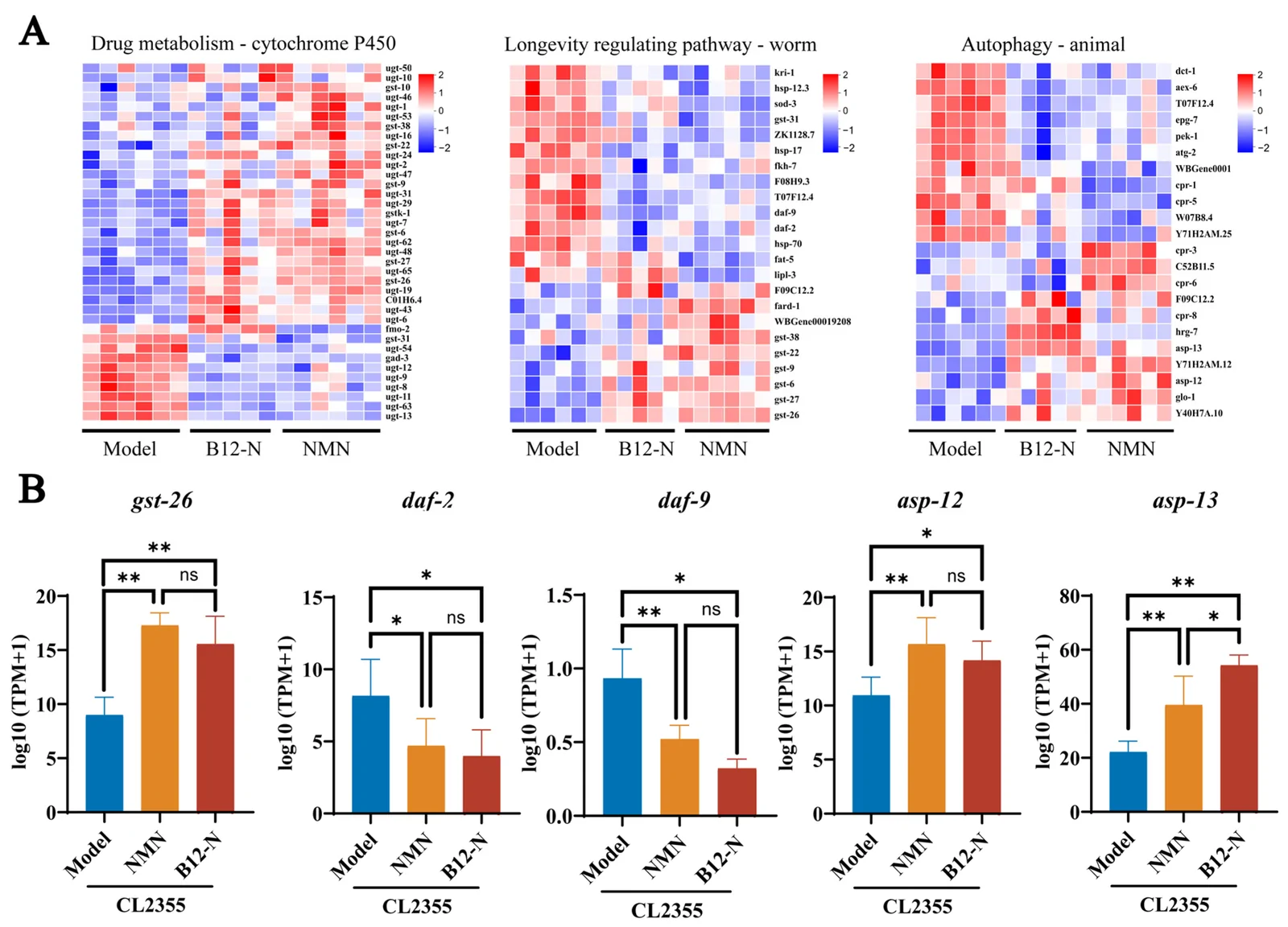
Heatmaps of enriched genes in the three pathways and representative genes. (**A**) Heatmaps of genes in the P450 metabolic pathway, longevity regulation pathway and autophagy pathway in CL2355 nematodes from the NMN and B12-N group. (**B**) Key genes in the P450 metabolic pathway, longevity regulation pathway and autophagy pathway. Three replicates per group; n > 1000 nematodes per replicate; for panel B, the data are presented as mean ± SD (ns, not significant, * *p* < 0.05, ** *p* < 0.01).

A cluster analysis was conducted on the genes within these three pathways. Based on the existing literature, five representative genes were selected for further analysis (Figure 9B). In the P450 metabolic pathway, the *gst-26* gene, which plays a role in oxidative stress and detoxification, was significantly up-regulated following interventions with NMN and B12-N (*p* < 0.01), with no significant difference observed between the two treatments (*p* > 0.05). In the longevity regulation pathway, the *daf-2* and *daf-9* genes, which are negatively correlated with lifespan, were significantly down-regulated following B12-N and NMN interventions (*p* < 0.05), with no significant difference between the two treatments (*p* > 0.05). Within the autophagy pathway, the *asp-12* and *asp-13* genes, which are involved in protein degradation and processing during autophagy, were significantly up-regulated following B12-N and NMN interventions (*p* < 0.05), the upregulation of asp-13 was significantly greater in the B12-N group than in the NMN group (*p* < 0.05).

## 4. Discussion

AD, the most prevalent neurodegenerative disorder, is characterized by progressive cognitive decline and is intimately linked to aging, which represents its greatest risk factor [53,54]. A key metabolic alteration in AD is the age-related decline in NAD+ [55], a crucial coenzyme for cellular energy metabolism and DNA repair, whose depletion is associated with core AD pathologies such as Aβ-associated amyloid aggregates and mitochondrial dysfunction [56]. While exogenous NMN supplementation can boost NAD+ levels and has shown promise in mitigating age-related decline [57], its clinical application is hampered by low bioavailability and high cost. Consequently, the potential of natural microbial sources, particularly lactic acid bacteria, to serve as live biotherapeutic factories for sustained in situ NMN production remains largely unexplored in the context of AD.

To address this gap, we screened 633 candidate strains by genomic pathway analysis, fluorometric screening, and LC–MS analysis, chemiluminescence assays and LC-MS analysis. An NMN-producing strain, *Lgb. salivarius* B12-N, was identified, with a measured cell-associated NMN content of 6.25 μg per 10^9^ CFU. Subsequently, using a transgenic *C. elegans* AD model expressing human Aβ_1–42_, NMN-producing probiotic strain B12-N and a content-matched dose of NMN were administered as drug interventions. Our study demonstrated that oral administration of B12-N significantly extends mean lifespan, and ameliorates Aβ-induced behavioral deficits. These beneficial effects are correlated with enhanced autophagy, reduced oxidative stress, and the suppression of Aβ-associated amyloid aggregates. The subsequent discussion will delve into the mechanisms by which B12-N orchestrates these pleiotropic outcomes, specifically its governance of pivotal signaling cascades—including the P450 metabolic axis, longevity regulation networks, and autophagic processes—and elucidate the broader ramifications for engineering probiotic therapeutics to combat AD [58].

The production of NMN by bacterial strains has attracted considerable academic interest due to its potential health benefits and applications in functional foods. A study examining NMN production by non-recombinant strains of *Limosilactobacillus reuteri* underscores the potential role of lactic acid bacteria (LAB) in this field. The research identified *L. reuteri* JCM 1112T as a producer of NMN, with production yields enhanced through co-cultivation with NAD+ synthesizing bacteria [59]. By identifying and overexpressing a native niacin transporter, lp2514, researchers achieved a significant increase in NMN production, thereby highlighting the potential of probiotics as a platform for NMN biosynthesis [60]. This approach is consistent with the broader trend of utilizing engineered microorganisms for sustainable NMN production [61]. These findings underscore the inherent capacity of LAB to naturally synthesize NMN. In this study, we analyzed the NMN production pathways in 633 strains of lactic acid bacteria derived from long-lived individuals using whole-genome data. The results demonstrated that nearly all lactic acid bacteria in this study synthesize NMN primarily through the de novo synthesis pathway. Subsequently, using fluorimetric and LC-MS assays, we quantitatively compared the NMN production capabilities of the strains and ultimately screened a strain of *Lgb. salivarius*, which exhibited significantly higher intracellular NMN accumulation levels compared to the other strains tested [31].

The potential of probiotics to alleviate AD and delay the aging process has gained increasing recognition. However, the intricate relationship between probiotics, NMN production, and their combined effects on AD and aging remains inadequately understood. Recent research has investigated the influence of NMN supplementation on gut microbiota and its potential therapeutic implications for AD. For example, NMN synbiotics, which integrate NMN with probiotics such as *Lgb. salivarius*, have been shown to restructure gut microbiota and modify metabolic pathways in AD mouse models [20]. Furthermore, NMN supplementation alone has been demonstrated to modulate gut microbiota and metabolites, thereby mitigating AD pathology in APP/PS1 mice [57]. We therefore conclude that there is a certain association between NMN and the ability of B12-N to alleviate defects in chemotaxis and the signs of aging [62].

Furthermore, transcriptomic analysis was performed to explore molecular pathways potentially associated with the effects of NMN and B12-N. Among the top 20 enriched pathways identified in each treatment group, 16 were shared between the NMN and B12-N groups, indicating partial overlap in their transcriptomic responses. Notably, pathways related to P450 metabolism, longevity regulation, and autophagy were significantly enriched. Notably, the P450 metabolism pathway, longevity regulation pathway, and autophagy pathway were significantly enriched.

Enhanced autophagy activity can delay aging and accelerate the clearance of Aβ and tau, thereby alleviating the pathology and symptoms associated with aging and AD. For example, rapamycin exerts anti-aging effects by inhibiting mTOR activity, thereby extending lifespan and healthy lifespan in mice [63]. Consequently, there is growing interest in enhancing the body’s autophagy activity to delay aging and in the protective role of autophagy in aging and age-related neurodegenerative diseases [63]. In this study, we utilized the transgenic *C. elegans* strains BC12921 and DA2123 to investigate the effects of NMN and probiotics on autophagy activity; these strains are commonly used to assess autophagy in *C. elegans*. The results indicated that NMN and probiotics significantly reduced SQST-1::GFP accumulation and increased LGG-1::GFP puncta. These concurrent changes are consistent with the modulation of autophagy-related processes and may reflect enhanced autophagic activity.

The *asp-12* and *asp-13* genes in the autophagy pathway (encoding aspartic proteases, which belong to the lysosomal protease family) have been shown to participate in protein degradation, lysosomal function and the regulation of autophagy flux; their upregulation in longevity models or neurodegenerative disease transcriptomes can enhance the clearance of misfolded proteins (such as Aβ), maintain protein homeostasis and alleviate neurotoxicity [64]. Together, these transcriptomic findings indicate that NMN and B12-N induce partially overlapping molecular responses involving pathways related to P450 metabolism, longevity regulation, and autophagy. These shared transcriptomic features are consistent with the possibility that the two interventions engage some common biological processes associated with aging and AD-related phenotypes.

P450 refers to the cytochrome P450 enzyme family, which is primarily involved in the metabolism of exogenous substances, detoxification and the synthesis of endogenous compounds [65]. Previous studies have reported enrichment of P450-related pathways in transcriptomic analyses of longevity interventions in *C. elegans*, often accompanied by altered expression of glutathione S-transferase (GST) genes involved in oxidative-stress defense and detoxification [66,67]. The gst-9 and *gst-26* genes within the P450 pathway have been shown to possess antioxidant, detoxifying and electrophile-scavenging functions; their upregulation in multiple RNA-seq studies alleviates environmental stress, improves age-related decline and promotes healthy longevity [68,69].

The *daf-2* and *daf-9* genes are established components of conserved longevity-regulatory pathways in *C. elegans*. *daf-2* encodes the insulin/IGF-1 receptor, and reduced DAF-2 signaling can promote nuclear activation of the downstream DAF-16/FOXO transcription factor and enhance the expression of genes involved in stress resistance and longevity [70]; *daf-9* encodes a specific CYP enzyme (CYP22A1), which participates in the synthesis of the dafachronic acid steroid hormone; regulates the activity of the DAF-12 nuclear receptor; and influences development, stress resistance and lifespan [71].

Aging is closely linked to AD, and growing evidence indicates that aging is a major risk factor for the development of neurodegenerative disorders [72]. Consequently, we investigated the anti-aging effects of NMN and probiotics in *Caenorhabditis elegans*. The results indicate that NMN and probiotics can significantly extend the average lifespan of CL2355 nematodes. Furthermore, age-related phenotypes, such as pharyngeal pump rate and motility, have been shown to decline as *C. elegans* age [73]. Together with the transcriptomic changes observed in longevity and stress-related pathways, these findings suggest that NMN and B12-N may influence multiple biological processes associated with age-related functional decline.

Although B12-N exhibits a high capacity for NMN production, and purified NMN displays similar protective phenotypes, the current experimental design does not prove that NMN is the specific or sole mediator of the beneficial effects of B12-N. In previous studies, we found that, due to the presence of alternative pathways, we were unable to generate a completely NMN-deficient B12-N derivative by disrupting genes involved in NMN biosynthesis. The strain B135U, which has low NMN production capacity, showed no significant difference from the model group in chemotaxis experiments and autophagy assays, but exhibited significant differences compared to the B12-N group. Furthermore, due to metabolic differences between strains, we were unable to use certain strains with low NMN production as negative controls to demonstrate that the effects of B12-N are largely attributable to NMN. Consequently, our findings support the notion that NMN is a potential contributing factor to the protective effects of B12-N, rather than establishing it as the sole causal mediator. Future research should utilize isogenic B12-N derivative in which the ability to biosynthesise NMN has been genetically knocked out, in order to directly determine the causal role played by bacterially produced NMN. Although B12-N contains substantial amounts of NMN before administration, the extent to which NMN is released from ingested bacterial cells, remains stable, and becomes available to the host has not been determined. Future studies directly quantifying NMN in the intestinal contents or whole-body extracts of *C. elegans*, together with measurements of NMN release from B12-N during feeding, will be necessary to determine whether B12-N provides NMN through continued bacterial production, release of pre-existing intracellular NMN, or both.

In summary, NMN and B12-N alleviated several AD-related and aging-associated phenotypes in the transgenic *C. elegans* CL2355 model. Transcriptomic analysis revealed partially overlapping changes in pathways related to P450 metabolism, longevity regulation, and autophagy following the two interventions. Together with the observed improvements in autophagy-related phenotypes, ThT-positive Aβ-associated amyloid-like aggregate burden, ROS levels, cognitive function, and lifespan, these findings suggest that NMN and B12-N may influence multiple biological processes associated with AD-related pathology and aging.

## Data Availability

The original contributions presented in this study are included in the article/Supplementary Material. Further inquiries can be directed to the corresponding authors.

## Supplementary Materials

The following supporting information can be downloaded at https://www.mdpi.com/article/10.3390/foods15203657/s1, Figure S1: Pathways of the nicotinic acid and nicotinamide metabolic pathway (map00760) from the publicly available KEGG database; Figure S2: NMN (nicotinamide mononucleotide) standard was analyzed using LC–MS.
